# *In Vitro* Susceptibility of* Mycobacterium abscessus* and* Mycobacterium fortuitum* Isolates to 30 Antibiotics

**DOI:** 10.1155/2018/4902941

**Published:** 2018-12-30

**Authors:** Yaojie Shen, Xuyang Wang, Jialin Jin, Jing Wu, Xuelian Zhang, Jiazhen Chen, Wenhong Zhang

**Affiliations:** ^1^Department of Infectious Diseases, Huashan Hospital, Fudan University, Shanghai, China; ^2^State Key Laboratory of Genetic Engineering, School of Life Science, Fudan University, Shanghai, China

## Abstract

**Objective:**

Nontuberculous mycobacteria (NTM) cause various diseases in humans and animals. Recently, the prevalence of NTM-related disease has been on the rise, becoming an emerging public health problem. The aim of this study was to determine the antibiotic susceptibility profiles of clinical isolates of* Mycobacterium abscessus and Mycobacterium fortuitum. Methods*. We performed susceptibility tests on 37 clinical NTM isolates to 30 antibiotics with the microdilution method recommended by the Clinical and Laboratory Standards Institute.

**Results:**

Both* M. abscessus* and* M. fortuitum* were highly resistant to antitubercular drugs such as isoniazid, rifampin, ethambutol, clofazimine, ethionamide, and rifabutin.* M. abscessus* showed the lowest resistant rates to cefoxitin (10%), azithromycin (10%), amikacin (10%), and clarithromycin (20%) and very high resistant to sulfamethoxazole, vancomycin, oxacillin, clindamycin, and all fluoroquinolones.* M. fortuitum* showed low resistance to tigecycline (0%), tetracycline (0%), cefmetazole (12%), imipenem (12%), linezolid (18%), and the aminoglycosides amikacin (0%), tobramycin (0%), neomycin (0%), and gentamycin (24%).

**Conclusion:**

Amikacin, cefoxitin, and azithromycin have the highest* in vitro* activity against* M. abscessus*. Isolates of* M. fortuitum* need to be individually evaluated for drug susceptibility before choosing an effective antimicrobial regimen for treatment of infections.

## 1. Introduction

Nontuberculous mycobacteria (NTM) are widely distributed in nature [1] and are opportunistic pathogens that can cause various diseases in multiple organs in humans and animals. Recently, the prevalence of NTM diseases has been on the rise [2, 3], and they are now recognized as representing an emerging public health problem [4].

*Mycobacterium abscessus* and* Mycobacterium fortuitum* are the most important RGMs (rapidly growing mycobacteria), with the former accounting for 80% of chronic pulmonary diseases caused by all RGM [5] and the latter being the main RGM responsible for extra-pulmonary disease, especially in cutaneous and plastic surgery-related infections [6].

*M. abscessus *is an opportunistic pathogen, which can cause human to human infection [7] and nosocomial infection [8]. It was reported to cause multiple community outbreaks of cutaneous infection by means of wading pool or swimming pool [9–11]. In addition,* M. abscessus* is one of the most severe drug resistant bacteria among the RGM [6, 12] and is therefore very difficult to treat [5, 13]. In order to achieve the goal of 12-month sputum conversion on medication, it is essential to guide treatment regimens based on drug susceptibility results [14].

*M. fortuitum* can often cause soft tissue infection during trauma and surgery. It had also been reported in many implant-associated infections [15] and in endocarditis infections [15, 16]. It was reported susceptible to multiple drugs except for macrolides [17]. However, the antibiotics resistance spectrum varies with different geographic locations or different hospital administration situation.

In this study, we investigated the drug susceptibility status of 30 commonly used antibiotics among 37 clinical isolates of* M. abscessus* and* M. fortuitum*. In addition, we compared the drug susceptibility results with those of other studies worldwide to provide a clearer picture of the current antibiotic resistance levels for these two common RGM species, which could serve as valuable reference data to guide treatment.

## 2. Methods

### 2.1. Strains and Antibiotics

In total, 20* M. abscessus* and 17* M. fortuitum* isolates were collected from various clinical specimens, including 23 sputum isolates, 5 bronchoalveolar lavage isolates, 2 puncture fluid isolates, 2 tissue isolates, 2 urine isolates, 1 wound isolate, 1 cerebrospinal fluid isolate, and 1 exudate isolate, from patients at the Huashan Hospital affiliated to Fudan University between January 2009 and December 2013. All isolates were from unique patients, except that one isolate of* M. abcessus* and* M. fortuitum* were cultured from two specimens of the same patient. All 37 isolates were recovered in Mueller-Hinton Broth (Oxoid, Hampshire, UK).* M. abscessus* ATCC19977 and* M. fortuitum* ATCC6841 were used as the reference strains.* Mycobacterium peregrinum* ATCC700686 was used as the quality control strain in drug susceptibility tests.

Rifampin, ethambutol, streptomycin, kanamycin, amikacin, ethionamide, clarithromycin, doxycycline, imipenem, linezolid, tobramycin, clindamycin, sulfamethoxazole, clofazimine, minocycline, neomycin, tetracycline, gentamycin, and vancomycin were purchased from Sigma-Aldrich (St. Louis, MO, USA). Isoniazid, levofloxacin, moxifloxacin, rifabutin, cefoxitin, ciprofloxacin, and oxacillin were purchased from Fluka/Sigma-Aldrich (St. Louis, MO, USA). Azithromycin, tigecycline, teicoplanin, and cefmetazole were purchased from Aladdin (Shanghai, China), Calbiochem/Sigma-Aldrich (St. Louis, MO, USA), Bio Vision (CA, USA), and Meilunbio (Dalian, China), respectively.

### 2.2. Subspecies Identification among M. abscessus Complex Isolates

Isolates were thawed and recovered on Lowenstein-Jensen medium at 37°C or on BACTER MGIT 960 medium for 4–7 days. DNA was extracted from cultured colonies using DNeasy Blood & Tissue Kit (Qiagen, Hilden, Germany) in accordance with the manufacturer's protocol and used as templates for polymerase chain reaction (PCR). The* rrs* gene [38] was amplified with primers* rrs*-F (5′-AGTTTGATCCTGGCTCAG) and* rrs*-R (5′-GGTTACCTTGTTACGACTT) and* hsp65*[18, 19, 39, 40] was amplified with primers* hsp*-F (5′-CGATGCGGTAAAGGTGACATTG) and* hsp*-R (5′-CCTTGACAGTGGACACCTTGGA). PCR was carried out in a final volume of 50 *μ*l with 1 *μ*l of DNA supernatant containing approximately 10 ng of genomic DNA, 5 *μ*l of 10× ExTaq PCR Buffer, 4 *μ*l of dNTPs (2.5 mM each), 0.4 *μ*M of each primer, 0.5 *μ*l of ExTaq DNA Polymerase (5 U/*μ*l) (Takara, Japan), and 37.5 *μ*l of distilled water. DNA samples were first denatured completely by incubation at 95°C for 5 min and then amplified using 35 cycles of (i) denaturation at 95°C for 40 s, (ii) primer annealing at 58°C for 40 s, and (iii) elongation at 72°C for 1 min in a thermocycler. The PCR products were sequenced by the Sanger method. Consensus sequences for each isolate were assembled using Lasergene SeqMan II software (DNAStar, Inc., Madison, WI, USA).

### 2.3. Drug Susceptibility Testing

The susceptibility tests of all 37 isolates and the reference* M. peregrinum* ATCC700686 against 30 antibiotics were carried out by the broth microdilution method in 96-well plates (Nunc, Denmark) according to the Clinical and Laboratory Standards Institute (CLSI) guidelines M24- A2. [41]. The final drug concentrations tested are shown in Table 1. The minimum inhibitory concentrations (MICs) of all antibiotics except for clarithromycin were determined after 3 days of incubation at 37°C. For clarithromycin, the incubation process lasted for 14 days. The MIC was determined as the lowest concentration of the drug that resulted in no visible bacterial growth. MIC90 values were defined as drug concentrations that inhibited 90% of the isolates. The susceptibility was determined based on CLSI breakpoint recommendations and published studies (Table 2). The resistance rates comparison between* M. abscessus *subsp.* abscessus* and* M. abscessus* subsp.* massiliense* was statistically analyzed by Fisher's exact chi-square test. A p-value of < 0.05 indicated statistical significance.

## 3. Results

### 3.1. Species Identification

There were 17 isolates identified as* M. fortuitum* and 20 isolates identified as* M. abscessus.* Among 20* M. abscessus *isolates, 12 isolates were classified as* M. abscessus *subsp.* abscessus*, 7 isolates were classified as* M. abscessus* subsp.* massiliense*, and one isolate was classified as* M. abscessus* subsp.* bolletii* based on their* hsp65* gene.

### 3.2. Resistance to Antitubercular Drugs

Both* M. abscessus* and* M. fortuitum* were highly resistant to antitubercular drugs such as isoniazid, rifampin, ethambutol, clofazimine, ethionamide, and rifabutin. Specifically, all isolates (100%) were resistant to isoniazid, ethambutol, and rifampin (Table 1). All of the* M. abscessus* isolates (100%) and 16 of the 17 (94%)* M. fortuitum* isolates were resistant to both ethionamide and rifabutin. Only one* M. abscessus* subsp.* abscessus* isolate and 7 of 17 (41%)* M. fortuitum* isolates were susceptible to clofazimine. This result reconfirmed that first-line antitubercular drugs are not useful for treating infections by* M. abscessus* or* M. fortuitum*. In addition, in order to better investigate the susceptibility of second-line antitubercular drugs to these strains, such as clofazimine, linezolid, and kanamycin, the first-line antitubercular drugs were partially used as a control in the study.

### 3.3. Resistance of M. abscessus to Non-Antitubercular Antibiotics

In general, the antibiotic resistance rates of the 20* M. abscessus* isolates were very high. All or almost all isolates were resistant to sulfamethoxazole, vancomycin, oxacillin, clindamycin, and all fluoroquinolones, and more than 50% of the isolates were resistant to tetracyclines, carbapenems, and aminoglycosides except for amikacin (Table 1).* M. abscessus* showed the lowest resistance rates to cefoxitin (10%), azithromycin (10%), amikacin (10%), and clarithromycin (20%). For clarithromycin, the 14-day inducible resistance rate is 15% (3/20). There was no statistically difference in the resistance rates between* M. abscessus* subsp.* abscessus* and subsp.* massiliense* isolates except to minocycline, in which the latter subspecies showed less resistance (Table 1).

### 3.4. Resistance of M. fortuitum to Non-Antitubercular Antibiotics

Although macrolides and cefoxitin are important component of the treatment regimen for RGM, the results showed that 16 (94%), 17 (100%), and 15 (88%)* M. fortuitum* isolates were resistant to clarithromycin, azithromycin, and cefoxitin, respectively. Besides macrolides, all or almost all of the* M. fortuitum* isolates were resistant to kanamycin (94%), doxycycline (82%), minocycline (82%), vancomycin (100%), teicoplanin (100%), oxacillin (100%), and clindamycin (100%).* M. fortuitum* showed the lowest levels of resistance to tigecycline (0%), tetracycline (0%), cefmetazole (12%), imipenem (12%), linezolid (18%), and the aminoglycosides, including amikacin (0%), tobramycin (0%), neomycin (0%), and gentamycin (24%) (Table 1). Isolates of* M. fortuitum* showed intermediate resistance rates to fluoroquinolones, which are also commonly considered in RGM treatment regimens. The resistance rates to moxifloxacin, ciprofloxacin, and levofloxacin were 59%, 47%, and 41%, respectively (Table 1). Importantly, the isolates showed variable resistance to three tetracyclines, tigecycline, and two cephalosporins. They were mostly resistant to doxycycline, minocycline, and cefoxitin but were susceptible to tigecycline and cefmetazole.

## 4. Discussion

Although the role of* in vitro* drug susceptibility testing has not been validated for most NTM species, these tests may nevertheless be important in the management of NTM-related diseases [19]. The need for long-term antibiotic treatment and associated toxicities contribute to the frequent unsatisfactory treatment outcomes in patients, thereby posing a great challenge to physicians in choosing the optimal regimen for patients infected with RGM. The present results of antibiotic susceptibility tests of the 30 most commonly used antibiotics against 37 RGM isolates, including subspecies of the* M. abscessus* complex isolates, supported the current recommendation of using amikacin, cefoxitin, and macrolides to treat* M. abscessus* infections [14]. Among the macrolides, azithromycin appears to be a better choice for treating* M. abscessus* infections than clarithromycin, since the susceptibility rate was lower for clarithromycin and the inducible resistance rate is 15%(3/20).

All* M. abscessus* isolates were resistant to streptomycin in our study (100%), which was in agreement with previous study [26]. However, in a study in Japan [30], the resistance rate to streptomycin was only 61%, which indicated the variance of the resistance in different healthcare background. For other rarely used aminoglycosides like tobramycin, neomycin, kanamycin, and gentamycin, the resistance rates of* M. abscessus* were also very high. The resistance rate to tobramycin was 60% in this study, which is higher than those of studies in Taiwan province of China [33], Korea [32], and Japan [30], in which the resistance rate varies from 30% to 32%. Broad spectrum antibiotics like minocycline, doxycycline, and sulfamethoxazole were ineffective against* M. abscessus* isolates; for their resistance rates reached 70%, 80%, and 90%, respectively. A recent study by Ruth et al. [42] proposed that minocycline has no clear roles in the treatment of* M. abscessus* disease, because of their high MICs against minocycline, rapid emergence of drug resistance, and no synergy effect with other antibiotics used to treat* M. abscessus*. Therefore, these antibiotics should not be used in clinics against* M. abscessus* infections.

Treatment with linezolid also appears to be a potentially good choice for this bacillus. The major difference between* M. abscessus* subsp.* abscessus* and* M. abscessus* subsp.* massiliense* is that the former has an innate* erm*(41) gene that confers the ability for inducible macrolide resistance; therefore, precise differentiation between these subspecies has been proposed to be important for clinical purposes [40, 43]. Our study showed that the macrolides resistance rate of* M. abscessus* subsp.* abscessus* was higher than that of* M. abscessus* subsp.* massiliense*, but the difference was not statistically significant due to the limitation of isolate numbers, which was consistent with the results conducted by Nie et al. [40]. However, except for macrolides and minocycline, differentiation between these two subspecies could not provide additional drug resistance information, as their resistance profiles were largely similar. In addition, the results of our study indicate that two commonly used drugs, macrolides and cefoxitin, should not be used in the treatment of* M. fortuitum* infection, since the isolates showed high resistance rates to both drugs. Given the lower resistance rates, aminoglycosides, cefmetazole, tigecycline, imipenem, and linezolid are potentially good choices for treatment regimens against* M. fortuitum* infection.

When the drug resistance rates obtained in the present study were compared with those reported previously, we found that high resistance to multiple antimicrobials was mostly prevalent in* M. abscessus*. Almost all studies indicated that* M. abscessus* isolates are highly susceptible to amikacin, cefoxitin, and macrolides and low resistance to linezolid (Table 3). By contrast, the imipenem data varied markedly among studies. The present study and those conducted in 2016 in Shanghai [24], 2017 in Taiwan [21], and 2015 in Australia [28] showed high resistance of* M. abscessus *to imipenem, whereas studies conducted in 2017 in Korea [22], 2013 in Guangdong [31], 2014 in Beijing [4], 2008 in Korea [32], and 2003 in Taiwan [33] showed relatively lower resistance rates to imipenem. Most studies did not support the use of fluoroquinolones and doxycycline, except for tigecycline (Table 3).

Although the drug resistance of* M. fortuitum* does not appear to be as prevalent as that of* M. abscessus*, the resistance rates to multiple antibiotics, including sulfamethoxazole, linezolid, clarithromycin, cefoxitin, and tobramycin, vary extensively among studies (Table 4). In addition, except for the present study, amikacin was reported to show the most effective* in vitro* activity in all previous studies. Most studies recommended the use of imipenem, except for the study conducted in 2017 in Guangzhou [34], in the treatment of* M. fortuitum* infection (Table 4). Low to medium degrees of fluoroquinolones resistance were observed in* M. fortuitum *in the majority of studies, in which moxifloxacin resistance varied from 0% to 59% and ciprofloxacin resistance varied from 3% to 47% (Table 4). The variability of susceptibility to different antimicrobials among studies emphasizes the importance of drug susceptibility testing in cases of* M. fortuitum* infection. The antibiotic susceptibility variance may be due to the difference in the choices of DST methods and breakpoints.

For some rarely used antibiotics applied in* M. fortuitum* treatment like neomycin and tigecycline, the susceptibility rates were 100% and 94% separately, suggesting they are very prospective antibiotics in the treatment. For gentamycin, although the susceptibility was only 24%, the intermediate rate was as high as 52%, which should be used in cautions in high doses.

Among the 36 patients, 29 were hospitalized and had detailed record of hospitalization. Among them, 27 have empirical antibiotic treatment, including meropenem, levofloxacin, clarithromycin, and rifampin, before the diagnosis of NTM associated diseases. Only 2 patients had no history of antibiotics treatment. Mostly patients had empirical anti-infection therapy in the hospital which could be the reason of the generally higher resistance rates in these isolates.

Nevertheless, this study has several limitations that should be mentioned. First, the isolates were geographically limited to a single province. Second, there was a limited number of RGM isolates collected, because of the relatively low incidence of RGM infections in Shanghai. Consequently, the power for detecting resistance to multiple antibiotics could be reduced in terms of generalizability to other RGM isolates worldwide.

In conclusion, the present results showed that amikacin, cefoxitin, and azithromycin had the highest* in vitro* activity against* M. abscessus*, which is in line with current recommendations. However, in contrast to previous studies,* M. fortuitum* was found to be mostly resistant to macrolides and cefoxitin. Therefore, isolates of* M. fortuitum* should be individually evaluated with the drug susceptibility test in deciding the most effective antimicrobials for treatment of infections.

## Figures and Tables

**Table 1:** The drug susceptibility results of 37 RGM isolates.

Antimicrobial Agent	MIC(*μ*g/ml)		*M. abscessus* (n=20)			MIC(*μ*g/ml)		*M. fortuitum* (n=17)		
	range	MIC 90	R	I	S	range	MIC 90	R	I	S
Macrolides										
CLR	0.0625-64	32	4 (20%)/7(35%)	0 (0%)/0 (0%)	16 (80%)/13(65%)	32-64	32	16 (94%)/17(100%)	0 (0%)/0 (0%)	1 (6%)/0(0%)
AZM	0.125-128	2	2 (10%)	0 (0%)	18 (90%)	8-128	32	17 (100%)	0 (0%)	0 (0%)
Rifamycins										
RFB	0.5-8	4	15 (75%)	-	5 (25%)	1-8	4	16 (94%)	-	1 (6%)
RIF	64-256	128	20 (100%)	-	0 (0%)	32-256	128	17 (100%)	-	0 (0%)
Aminoglycosides										
STR	32-128	64	20 (100%)	-	0 (0%)	16-128	32	17 (100%)	-	0 (0%)
GEN	0.5-64	32	14 (70%)	1 (5%)	5 (25%)	4-64	32	4 (24%)	9 (53%)	4 (24%)
KAN	4-16	8	16 (80%)	-	4 (20%)	4-32	16	16 (94%)	-	1 (6%)
TOB	2-32	8	12 (60%)	3 (15%)	5 (25%)	16-64	16	0 (0%)	0 (0%)	17 (100%)
NEO	0.5-64	16	13 (65%)	-	7 (35%)	2-8	2	0 (0%)	-	17 (100%)
AMK	0.5-64	32	2 (10%)	9 (45%)	9 (45%)	2-8	4	0 (0%)	0 (0%)	17 (100%)
Fluoroquinolones										
MXF	0.0625-16	8	19 (95%)	0 (0%)	1 (5%)	0.0625-8	2	10 (59%)	5 (29%)	2 (12%)
CIP	0.125-128	64	19 (95%)	0 (0%)	1 (5%)	0.125-256	128	8 (47%)	0 (0%)	9 (53%)
LVX	0.125-32	16	19 (95%)	0 (0%)	1 (5%)	0.125-32	16	7 (41%)	0 (0%)	10 (59%)
Cephalosporins										
FOX	16-256	64	2 (10%)	10 (50%)	8 (40%)	16-128	32	15 (88%)	0 (0%)	2 (12%)
CMZ	2-256	128	11 (55%)	5 (25%)	4 (20%)	2-64	8	2 (12%)	0 (0%)	15 (88%)
Tetracyclines										
TCY	2-256	64	9 (45%)	3 (15%)	8 (40%)	1-8	4	0 (0%)	12 (70%)	5 (30%)
DOX	0.5-256	128	14 (70%)	2 (10%)	4 (20%)	0.5-256	128	15 (88%)	0 (0%)	2 (12%)
MNO	0.125-128	64	11 (55%)	3 (15%)	6 (30%)	0.25-32	16	14 (82%)	1 (6%)	2 (12%)
Glycylcycline										
TGC	0.0625-16	8	8 (40%)	6 (30%)	6 (30%)	0.0625-4	0.5	0 (0%)	1 (6%)	16 (94%)
Sulfonamides										
SOX	2-256	128	18 (90%)	-	2 (10%)	16-256	128	14 (82%)	-	3 (18%)
Carbapenems										
IMP	1-256	64	13 (65%)	2 (10%)	5 (25%)	2-32	16	2 (12%)	12 (70%)	3 (18%)
Oxazolidinones										
LNZ	2-128	16	3 (15%)	1 (5%)	16 (80%)	8-32	16	3 (18%)	12 (70%)	2 (12%)
Lincosamides										
CLI	8-256	128	20 (100%)	0 (0%)	0 (0%)	256	256	17 (100%)	0 (0%)	0 (0%)
Penicillins										
OXA	256	256	20 (100%)	-	0 (0%)	256	256	17 (100%)	-	0 (0%)
Polypeptides										
TEC	0.5-256	128	17 (85%)	1 (5%)	2 (10%)	128-256	128	17 (100%)	0 (0%)	0 (0%)
VAN	256	256	20 (100%)	0 (0%)	0 (0%)	256	256	17 (100%)	0 (0%)	0 (0%)
Others										
CFZ	0.25-128	32	19 (95%)	-	1 (5%)	0.125-128	0.25	10 (59%)	-	7 (41%)
EMB	256	256	20 (100%)	-	0 (0%)	256	256	17 (100%)	-	0 (0%)
INH	8-256	16	20 (100%)	-	0 (0%)	8-16	8	17 (100%)	-	0 (0%)
ETH	16-256	64	20 (100%)	-	0 (0%)	4-256	32	16 (94%)	-	1 (6%)

(a) CLR= clarithromycin, AZM= azithromycin, RFB= rifabutin, RIF= rifampin, STR= streptomycin, GEN= gentamycin, KAN= kanamycin, TOB= tobramycin, NEO= neomycin, AMK= amikacin, MXF= moxifloxacin, CIP= ciprofloxacin, LVX= levofloxacin, FOX= cefoxitin, CMZ= cefmetazole, TCY= tetracycline, DOX= doxycycline, MNO= minocycline, TGC= tigecycline, SOX= sulfamethoxazole, IMP= imipenem, LNZ= linezolid, CLI= clindamycin, OXA= oxacillin, TEC= teicoplanin, VAN= vancomycin, CFZ= clofazimine, EMB= ethambutol, INH= isoniazid, and ETH= ethionamide.

(b) “-“ indicates data not available.

(c) For CLR, each resistance and susceptible rate has two data. The first is the MIC result for 3 days and the second is the MIC result for 14 days.

**Table 2:** Breakpoints of 30 antibiotics.

Antimicrobial Agent		MIC breakpoints (*μ*g/ml)		
		Susceptibility	Intermediate	Resistance
*Macrolides*	CLR^a^	⩽2	4	*⩾*8
	AZM^c^	⩽2	4	*⩾*8
*Rifamycins*	RIF^a^	-	-	>1
	RFB^a^	-	-	>2
*Aminoglycosides*	STR^a^	-	-	*⩾*5
	GEN^b^	<=4	8	>=16
	KAN^a^	-	-	*⩾*4
	TOB^a^	⩽2	4	*⩾*8
	NEO^b^	-	-	>=10
	AMK^a^	⩽16	32	*⩾*64
*Fluoroquinolones*	MXF^a^	⩽1	2	*⩾*4
	CIP^a^	⩽1	2	*⩾*4
	LVX^a^	⩽2	4	*⩾*8
*Cephalosporin*	FOX^a^	⩽16	32-64	*⩾*128
	CMZ^d^	⩽16	32	*⩾*64
*Tetracyclines*	TCY^b^	<=4	8	>=16
	DOX^a^	⩽1	2-4	*⩾*8
	MNO^a^	⩽1	2-4	*⩾*8
*Glycylcycline*	TGC^a^	⩽1	2-4	*⩾*8
*Sulfonamides*	SOX^a^	⩽38	-	*⩾*76
*Carbapenems*	IMP^a^	⩽4	8-16	*⩾*32
*Oxazolidinones*	LNZ^a^	⩽8	16	*⩾*32
*Lincosamides*	CLI^b^	<=0.5	1-2	>=4
*Penicillins*	OXA^b^	<=2	-	>=4
*Polypeptides*	TEC^b^	<=8	16	>=32
	VAN^b^	<=2	4-8	>=16
Others	CFZ^e^	-	-	>1
	INH^a^	-	-	*⩾*1
	EMB^a^	-	-	*⩾*4
	ETH^a^	-	-	>5

a denotes the breakpoints coming from **Susceptibility Testing of Mycobacteria, Nocardia, and Other Aerobic Actinomycetes; Approved Standard–Second Edition**. *CLSI document M24-A2*.

b denotes the breakpoints coming from Performance Standards Antimicrobial Susceptibility Testing-27th Edition.* CLSI document M100*.

c, d, e denote the breakpoints coming from [18–20], respectively.

d, S, susceptible, I, intermediate susceptible, and R, resistant.

**Table 3:** The comparison of drug resistance rate of *M*. *abscessus *isolates from various studies.

district/nation	year	Test method	Tetracyclines		Glycylcycline	Carbapenems					Aminoglycosides			Fluoroquinolones			Macrolides			source
			DOX	MNO	TGC	IMP	MEM	LNZ	SXT/SOX^3^	FOX	AMK	TOB	STR	CIP	MXF	LVX	CLR-ERT	CLR-LRT	AZM	
Shanghai	2017	1	14 (70%)	11 (55%)	8 (40%)	13 (65%)	-	3 (15%)	18 (90%)	2 (10%)	2 (10%)	12 (60%)	20 (100%)	19 (95%)	19 (95%)	19 (95%)	-	9 (45%)	2 (10%)	This study
Taiwan	2017	1	66 (99%)	66 (99%)	-	41 (61%)	-	47 (70%)	60 (90%)	14 (21%)	4 (6%)	-	-	60 (90%)	63 (94%)	-	5 (7%)	19 (28%)	-	[21]
Korea	2017	1	-	-	-	17 (18%)	-	5 (5%)	-	10 (10%)	6 (6%)	-	-	94 (99%)	88 (93%)	-	9 (9%)	39 (41%)	-	[22]
Beijing	2016	1	-	-	4 (18%)	9 (41%)	-	2 (9%)	10 (45%)	7 (32%)	1 (5%)	8 (36%)	-	-	6 (27%)	17 (77%)	3 (14%)	-	17 (77%)	[23]
Shanghai	2016	1	52 (98%)	-	-	52 (98%)	-	11 (21%)	-	15 (28%)	1 (2%)	44 (83%)	-	-	51 (96%)	-	15 (28%)	36 (68%)	-	[24]
UK	2015	2	-	-	3 (1%)	-	534 (99%)	311 (96%)	-	531 (96%)	74 (14%)	-	-	495 (99%)	257 (98%)	-	33 (6%)	-	191 (38%)	[25]
Fujian et al.	2015	1	36 (65%)	31 (56%)	2 (4%)	-	16 (29%)	2 (4%)	-	17 (31%)	0 (0%)	30 (55%)	52 (95%)	31 (56%)	12 (22%)	29 (53%)	18 (33%)	-	12 (22%)	[26]
Singapore	2015	1	235 (80%)	-	-	50 (20%)	-	50 (16%)	95 (94%)	8 (3%)	1 (0.9%)	282 (98%)	-	293 (94%)	115 (93%)	-	11 (3%)	-	-	[27]
Australia	2015	1	32 (84%)	-	-	26 (68%)	-	7 (18%)	35 (92%)	7 (18%)	2 (5%)	22 (58%)	-	36 (95%)	35 (92%)	-	-	18 (47%)	-	[28]
Beijing	2014	1	-	-	-	7 (10%)	-	2 (3%)	-	1 (1%)	1 (1%)	-	-	-	10 (14%)	68 (97%)	22 (31%)	38 (54%)	3 (4%)	[4]
Korea	2014	1	-	-	-	-	-	-	-	-	40 (10%)	-	-	361 (89%)	319 (79%)	-	64 (16%)	186 (46%)	-	[29]
Japan	2013	1	139 (97%)	119 (83%)	73 (51%)	34 (24%)	-	16 (11%)	143(100%)	-	7 (5%)	44 (31%)	87 (61%)	137 (96%)	135 (94%)	-	18 (13%)	-	-	[30]
Guangdong	2013	1	-	-	-	15 (21%)	-	-	-	3 (4%)	0 (0%)	-	-	56 (80%)	-	-	10 (14%)	-	-	[31]
Korea	2008	1	60 (81%)	-	-	8 (11%)	-	-	-	0 (0%)	0 (0%)	22 (30%)	-	13 (18%)	5 (7%)	-	2 (3%)	-	-	[32]
Taiwan	2003	1	85 (92%)	-	-	17 (10%)	91 (99%)	39 (42%)	91 (99%)	4 (4%)	4 (4%)	28 (32%)	-	87 (96%)	75 (82%)	88 (96%)	10 (11%)	-	44 (48%)	[33]

(1) 1 means broth microdilution method and 2 means disc diffusion method.

(2) “-“ means the results are not available.

(3) SXT=trimethoprim-sulfamethoxazole. SXT and SOX have the same mainly effective ingredient sulfamethoxazole, so we compare the results together.

**Table 4:** The comparison of drug resistance rate of *M. fortuitum* isolates from various studies.

district/nation	year	Test method	Aminoglycosides			Fluoroquinolones				Carbapenems			Sulfonamides	source
			AMK	TOB	FOX	MXF	CIP	CLR	DOX	IMP	MEM	LNZ	SXT/SOX^3^	
Shanghai	2017	1	0 (0%)	0 (0%)	15 (88%)	10 (59%)	8 (47%)	17 (100%)	15 (88%)	2 (12%)	-	3 (18%)	14 (82%)	This study
Guangzhou	2017	1	7 (14%)	51 (100%)	-	2 (4%)	21 (41%)	39 (76%)	-	29 (57%)	13 (25%)	43 (84%)	-	[34]
Mumbai	2016	2	0 (0%)	0 (0%)	22 (100%)	-	5 (23%)	0 (0%)	-	4 (18%)	-	0 (0%)	21 (95%)	[35]
India	2016	1	0 (0%)	-	-	-	5 (24%)	3 (14%)	-	-	-	-	-	[36]
Iran	2016	1	1 (2%)	0 (0%)	8 (14%)	25 (29%)	12(14%)	-	36 (42%)	8 (9%)	42 (49%)	6 (7%)	0 (0%)	[37]
Singapore	2015	1	3 (3%)	73 (92%)	7 (8%)	0 (0%)	3 (3%)	44 (47%)	-	3 (3%)		6 (7%)	1 (3%)	[27]
Taiwan	2003	1	0 (0%)	32 (46%)	1 (1%)	17 (25%)	23(33%)	14 (20%)	47 (68%)	5 (7%)	28 (41%)	17 (25%)	35 (51%)	[33]

(1) 1 means broth microdilution method and 2 means disc diffusion method.

(2) “-“ means the results are not available.

(3) SXT=trimethoprim-sulfamethoxazole. SXT and SOX have the same mainly effective ingredient sulfamethoxazole, so we compare the results together.

## Data Availability

The data used to support the findings of this study are included within the article.

## Abbreviations

MIC: Minimum inhibitory concentration
NTM: Nontuberculous mycobacteria
RGM: Rapidly growing mycobacteria.
